# Cases of Puerperal Jaundice

**Published:** 1802-10-01

**Authors:** 


### *To the Editors of the Medical and Physical Journal.*

Gentlemen,

I have met with ſeveral caſes of Puerperal Jaundice occurring in early pregnancy, that yielded to the moſt ſimple method of treatment; in advanced pregnancy we have, however, to fear its fatal effects, owing to the great weakneſs and relaxation of the ſyſtem attending this diſeaſe, which muſt be greatly increaſed by the debility accompanying parturition. The two following caſes are inſtances of the fatal tendency of Puerperal Jaundice; as I thought them ſingular, I have tranſcribed them from my journal book. If you think them worthy a place in your uſeful Journal, their inſertion will oblige your's &c.

*Aberdeen, August* 16, 1802. T. P.

---

### Case I.

I was ſent for to deliver B. F. about four o'clock in the morning; the room was on the ground floor, ſeemed very damp, and had a rotten ſmell, with a ſmall lamp burning which afforded but little light. I had not ſeen the woman before, and jaundice being a diſeaſe which is not ſo perceivable at night, although aſſiſted by a good light from candles, which was not the caſe here, I therefore did not diſcover that ſhe was labouring under this diſeaſe, till day light, notwithſtanding ſhe had the ſymptoms of it; but labour pains attending at the ſame time, my chief attention was paid to the latter. When I examined her her previous to delivery, ſhe ſaid ſhe had been for ſome time affected with ſickneſs and vomiting, loathing of food, pain in the epigaſtric region, and a ſhort cough. At my firſt arrival her labour-pains were gentle; ſhe was ſitting in a chair, and would not be prevailed upon to be delivered in her bed, obſerving that ſhe was always delivered out of bed with all her other children. In leſs than two hours from the time I firſt went there, ſhe was delivered; the labour was natural, the amnien tumour burſt of itſelf, the vertex preſented, and the delivery was very eaſy to the woman. The placenta was alſo very eaſily brought forth. After the woman was delivered, I went home; and in the morning when I again viſited her, I perceived her face, eyes, and whole body, very yellow; ſhe ſeemed however pretty eaſy, and ſpoke with a ſtrong voice; but did not pay much attention to any perſon around her. When aſked how ſhe felt herſelf, ſhe ſaid ſhe was tolerably eaſy, and had no complaint. She continued pretty eaſy till the evening of the ſecond day after delivery; when ſhe was ſeized with coldneſs and ſhivering, attended with incoherency of ſpeech. Next morning when I viſited her, ſhe was perfectly inſenſible, affected with ſtupor and coma, appearing as if ſhe were in a conſtant ſleep; her urine, which was of a deep yellow colour, ſhe voided inſenſibly; belly coſtive. She never opened her eyelids, and in that condition took whatever drink was given her; ſhe never aſked, however, for drink or any thing. Wine and other ſtimulant cordials were given her, which ſhe ſwallowed when put into her mouth. Her head was ſhaved, and a bliſter applied, which acted well, and contained a yellow lymph. All the medicines that were applied, had no effect, either in bringing her to her ſenſes, or removing the jaundice. A clammy ſweat continued on her ſkin from the time of her becoming inſenſible; her ſwallowing became more difficult; and a ſnorting attended. Her pulſe kept always from 70 to 75, till within a ſhort time of her death, which happened five days after her delivery.

### Case II.

I was called to I. H. on a Friday forenoon; ſhe was in bed; her ſkin was of a deep yellow colour; during my ſtay there, ſhe vomited a great quantity of brown ſtuff. The vomiting had been very frequent during the morning. She appeared to be torpid and inſenſible, with a quick and ſmall pulſe, and a ſhort tickling cough which ſeized her now and then. When I aſked what ſhe felt ſore or uneaſy to her, ſhe anſwered with a weak voice, that ſhe had a pain at her breaſt, and that her whole body was fore. I ordered her a weak cordial mixture, with a view to fettle the vomiting. I was not a quarter of an hour gone, when labour pains came on. The midwife was attending, and fhe was delivered of a child with a few pains. In half an hour after delivery, they fent for me again, faying, fhe was worfe. The vomiting had only feized her once; but the torpor and infenfibility were increafed. When I afked the midwife concerning her labour, fhe anfwered, that the patient appeared to have a defire to void her urine, and when fhe was affifting her, fhe found the child's head in the pelvis, which was delivered by a few pains. On Friday afternoon and evening fhe became ftill worfe, appearing to be afleep and quite infenfible. I applied a blifter to her neck, and ordered her to get plenty of wine; fhe, however, ftill grew worfe, and lived only till four o'clock on Saturday morning.

